# Cognitive alignment in cardiovascular AI: designing predictive models that think with, not just for, clinicians

**DOI:** 10.3389/fcvm.2025.1651324

**Published:** 2025-09-02

**Authors:** Jeena Joseph, K. Kartheeban

**Affiliations:** ^1^ Department of Computer Applications, Kalasalingam Academy of Research and Education, Krishnankoil, India; ^2^ Department of Computer Applications, Marian College Kuttikkanam Autonomous, Kuttikkanam, India; ^3^ Department of Computer Science and Engineering, Kalasalingam Academy of Research and Education, Krishnankoil, India

**Keywords:** cardiovascular disease, cognitive alignment, clinical decision support, explainable AI, human-AI collaboration, medical trust

## Introduction

Artificial intelligence (AI) is emerging as a major driver of clinical innovation, with cardiovascular disease (CVD) prediction being one of its most active areas of application (1, 2). In recent years, hospitals, research centers, and health-technology companies have reported machine learning models achieving accuracy levels of 90%, 95%, or even higher for predicting heart attacks, arrhythmias, and other cardiovascular events, with concrete evidence shown in studies on AI-enabled ECG detection of left ventricular dysfunction and machine learning-based outcome prediction in heart failure (3, 4). These results highlight the significant technical progress made in the field. Despite these encouraging statistics, adoption of AI tools in day-to-day cardiovascular practice remains limited (5). This raises a central question: if AI models demonstrate such high accuracy in controlled evaluations, why are they not widely used in clinical settings?

This study explores that question by moving beyond accuracy as the sole measure of success. While many cardiovascular AI models are developed with strong technical performance—demonstrating high discrimination, well-calibrated risk estimates, robustness to data shifts, and external validation—their adoption in practice also depends on how well their outputs integrate into clinicians' established workflows and support decision-making under real-world time constraints and uncertainty. Even when demonstrating strong algorithmic performance—such as high discrimination (e.g., AUC), well-calibrated risk estimates, robust external validation, and resilience to moderate data shifts—many current AI models still fail to integrate with the way clinicians gather, interpret, and apply information during patient care (6). These shortcomings often arise from interface design gaps, limited explainability, lack of EHR integration, and poor alignment with established clinical reasoning workflows. We refer to this gap as a lack of cognitive calibration—the degree to which AI tools reflect, augment, and support a clinician's reasoning process. To address this, we propose a Cognitive Alignment Index (CAI)—introduced here and detailed later—which evaluates models not only on statistical accuracy but also on comprehensibility, actionability, feedback receptivity, context awareness, and calibrated trust.

Addressing cognitive alignment requires a shift in focus. Instead of designing models solely to outperform human performance on test datasets, the goal should be to create systems that enhance clinical reasoning in explainable, interpretable, and contextually relevant ways (7, 8). For CVD applications, this means moving from automation toward augmentation—from opaque, black-box predictions to collaborative, co-reasoned decision-making (9).

## The missing link: cognitive alignment

Current debates regarding trustworthy AI tend to focus on concepts like explainability, fairness, and robustness. These are important, but they mainly address models' extrinsic properties. Cognitive alignment, instead, investigates an internally compatible human- machine style of reasoning integration (10). It asks: Do AI systems process and report information in such modes that clinicians will be in a position to intuitively comprehend, critique and act on?

We can define cognitive alignment as the degree to which an AI system's reasoning processes, information presentation, and interaction patterns correspond to and enhance the cognitive processes clinicians use in real-world decision-making. This concept spans five core constructs—narrative coherence, counterfactual reasoning, progressive disclosure, uncertainty communication, and interactive collaboration—each contributing to a shared human–AI decision framework. While it overlaps with concepts such as explainability, usability, trust calibration, and shared mental models, cognitive alignment is distinct in its focus on mutual intelligibility and collaborative reasoning between clinician and model.

Consider a 68-year-old with chest pain, discordant biomarkers and imaging, chronic kidney disease, diabetes, prior stroke, and limited access to follow-up care. The clinician must reconcile conflicting evidence, weigh competing risks, and factor in social constraints. A cognitively aligned AI could mirror this reasoning—integrating multimodal data, generating counterfactuals, and conveying calibrated uncertainty to guide a patient-specific decision.

Contrast this with a typical CVD risk prediction model. It may take in a static data set, calculate probabilistic risk, and provide an output—e.g., 0.87 probability of cardiovascular event at 5 years. The model reveals little regarding why this score is high, what modifiable variables impacted it, or in what way it changes in response to new interventions. Many static, black-box risk scoring models—especially those trained on structured tabular datasets—present outputs solely as numeric probabilities without contextual explanation, making them less intuitive for clinical reasoning (11). In contrast, well-designed systems, including those using case-based reasoning, counterfactual analysis, or natural-language generation, can produce narrative rationales and patient-specific explanations that align more closely with how clinicians synthesize information (12).

This mismatch is not abstract. It begets a disconnect in trust, usability, and accountability (13). Where an output from an AI raises doubt in a clinician's intuition, and no middle ground lies, the human decision-maker falls back to skepticism or dismissal. Worse, if clinicians over-rely on an opaque model, the result is over-reliance in faulty predictions—perilous in life-critical decisions (14). Recent empirical work underscores the importance of explainability and cognitive alignment. A systematic review of XAI in clinical decision support systems found that only a minority of applications formally evaluated explanation quality, highlighting trust as a critical, yet often underexamined, dimension (15, 16). Another study demonstrated that cardiovascular event forecasting systems augmented with XAI increased user comprehension and decision—making confidence, achieving both high accuracy and improved usability (17). These findings show that enhancements to interpretability directly improve trust and adoption—validating our argument that accuracy alone is not enough without cognitive alignment.

## Where current cardiovascular AI falls short

The limitations discussed in this section refer primarily to classes of cardiovascular AI models that are i. trained on static, cross-sectional datasets, ii. optimized for predictive accuracy rather than interpretability, and iii. deployed without advanced temporal modeling, multimodal integration, or narrative explanation capabilities. These constraints do not apply to all cardiovascular AI architectures—many state-of-the-art models in research settings already address some of these issues—but they remain common in tools currently used in routine clinical practice. In practice, widely deployed cardiovascular AI tools include FDA-cleared ECG algorithms for LV dysfunction (18), AI-guided echo acquisition (19), CT-FFR integrated into NHS pathways (20), and EHR-based HF readmission models. In contrast, research prototypes—such as multimodal transformers combining ECG, echo, and EHR data (21, 22) or counterfactual imaging interpreters—remain largely academic. Recent work has demonstrated their potential, including improvements in arrhythmia detection (23), development of patient digital-twin frameworks (24), and transformer-based atrial fibrillation risk prediction (25). Cognitive alignment gaps are most pronounced in deployed models, not these cutting-edge prototypes. The failure of many currently deployed cardiovascular AI models—particularly static tabular classifiers trained on cross-sectional datasets—to achieve cognitive alignment manifests in several critical areas:

• Temporal Reasoning Deficiency: Clinicians reason over time, comparing past trajectories to future projections. Many cardiovascular AI tools currently deployed in clinical settings—particularly static tabular classifiers trained on cross-sectional snapshots of EHR data—lack temporal reasoning capabilities. These models reduce patient history to single time points, overlooking evolving physiological trends that clinicians use for decision-making. This limitation does not apply to temporal sequence models such as RNNs, LSTMs, Transformers, temporal convolutional networks (TCNs), survival analysis models like Cox/DeepSurv, or dynamical Bayesian/state-space approaches, which are explicitly designed to learn from longitudinal data (26).
• Opaque Abstractions: Clinicians prefer causal or mechanistic reasoning—“this patient's sedentary lifestyle, combined with family history, likely explains the elevated risk.” In contrast, black-box models offer abstractions untethered from causal understanding (27). A high risk score may be mathematically correct, but without interpretive scaffolding, it remains clinically inert.
• Disjointed Input and Output Modalities: Doctors process multimodal data—lab results, imaging, voice tone, visual signs. Most AI models require clean, structured inputs and output a single prediction (28). This limits their ability to integrate into the messy, multimodal ecosystem of real clinical practice.
• Static Decision Boundaries: In some deployments, binary classifiers are paired with fixed probability thresholds (e.g., intervene if risk > X%), which is often a policy choice rather than an inherent model property. While such cut points can simplify implementation, they may overlook trade-offs, comorbidities, patient preferences, and evolving clinical information. More flexible approaches—such as continuous risk estimates, decision-curve analysis, and context-aware policies that adapt thresholds to individual patient contexts—better reflect the nuanced nature of cardiovascular decision-making.

It is important to acknowledge that recent advances in AI research have begun to address some of these limitations. Emerging models now incorporate temporal reasoning, causal inference, counterfactual simulation, and more sophisticated multimodal integration, enabling them to analyze evolving patient trajectories, draw connections between complex variables, and provide richer explanations (29–31). These capabilities represent a significant step forward and demonstrate the technical feasibility of overcoming many past shortcomings. However, their presence in cutting-edge research does not yet equate to widespread adoption in clinical cardiovascular tools. Many AI systems currently deployed in hospitals or available in commercial products still operate with static inputs, limited interpretability, and rigid decision boundaries (32, 33). Thus, while technical progress is undeniable, the challenge remains in translating these capabilities into routinely used systems that align seamlessly with clinicians' cognitive workflows and decision-making processes.

## Toward cognitively aligned cardiovascular AI

Cognitive alignment demands that we reframe how CVD prediction models are conceived, trained, and evaluated. Below are key dimensions that define such alignment:

• Narrative Coherence: AI outputs should tell a story. Instead of “risk = 0.87,” the model might say: “This patient's risk is elevated primarily due to high LDL, low physical activity, and a recent increase in blood pressure. Reducing any of these could lower the 5-year risk estimate.” This aligns with how clinicians communicate risk to patients and among themselves. Models must be able to generate semantically rich explanations—preferably in natural language (34). For example, reasoning-capable large language models can be integrated with structured learners such as XGBoost, using SHAP values or other feature attribution methods to generate narrative rationales that combine quantitative predictions with case-based, clinically meaningful explanations.
• Counterfactual Thinking: One hallmark of clinical reasoning is asking “what if?” What if the patient starts statins? What if they quit smoking? A cognitively aligned AI should support counterfactual queries**,** allowing clinicians to explore alternative scenarios. Some emerging models incorporate causal inference and counterfactual simulation (35). These should be further integrated into CVD AI to facilitate planning, not just prediction.
• Progressive Disclosure: Rather than flooding users with all data at once or hiding it entirely, models should offer layered explanations. An initial summary could be followed by options to “drill down” into data weights, feature contributions, or example-based analogies. This mirrors the way clinicians seek different levels of information depending on the urgency, context, and confidence level.
• Uncertainty as a Feature, not a Bug: Human decision-making in medicine is riddled with uncertainty. Some AI models—particularly those without proper calibration—may display high predicted probabilities even in cases of misclassification or when encountering out-of-distribution inputs. In contrast, well-calibrated models, such as logistic regression or modern neural architectures with calibration layers, can align predicted probabilities with actual likelihoods. A cognitively aligned model should quantify and communicate uncertainty, and offer calibrated risk intervals or confidence distributions (36). This enables clinicians to factor in model hesitancy into their broader clinical judgment.
• Interactive Collaboration: The future of CVD AI lies in interactive systems—not static dashboards, but conversational agents or co-pilot interfaces that let clinicians query, challenge, and modify model outputs in real time. These tools should learn not only from data, but from dialogue with human users.
• Measurement Framework: Each dimension of cognitive alignment can be operationalized through observable indicators that allow for systematic evaluation in both simulated and real-world clinical contexts. Narrative coherence can be assessed through comprehension scores, accuracy in summarizing reasoning chains, and retention of key factors. Counterfactual reasoning may be measured by the accuracy and clinical relevance of “what-if” scenarios and the frequency with which they lead to actionable plan adjustments. Progressive disclosure can be evaluated by reductions in decision latency without loss of accuracy and the successful retrieval of deeper model details on demand. Uncertainty communication may be gauged through the appropriateness of decision adjustments based on model confidence intervals and the reduction in over-reliance on low-certainty predictions. Finally, interactive collaboration can be quantified by the rate of appropriate overrides, the proportion of outputs refined through clinician feedback, and the interception of potential errors when human–AI disagreements occur.

## Framework for the cognitive alignment index (CAI)

The Cognitive Alignment Index (CAI) measures how well an AI system aligns with clinicians' reasoning across five dimensions: comprehensibility, actionability, feedback receptivity, context awareness, and trust calibration. Each dimension is linked to measurable indicators, enabling consistent evaluation and benchmarking.

Cardiovascular AI often integrates heterogeneous inputs—such as imaging, electronic health records (EHR), and wearable data—that are vulnerable to temporal misalignment, incomplete provenance tracking, and biased missingness. These multimodal integration challenges can be mitigated through alignment strategies, including progressive disclosure of modality-specific findings and saliency maps tailored to each data type.

Failures can originate from data (e.g., out-of-distribution inputs), model (e.g., overfitting, uncalibrated uncertainty), interface (e.g., unclear explanations), or workflow (e.g., poorly timed alerts). Mitigation measures include out-of-distribution detection, decision guardrails, and clinician override mechanisms with mandatory rationale capture.

For transparency and medico-legal defensibility, CAI recommends audit trails that log explanations shown, uncertainty estimates, and clinician actions or overrides with accompanying rationale. Finally, the framework distinguishes trust—belief in the system's competence—from reliance—acting on its output—and emphasizes calibrated trust, ensuring reliance remains proportional to the system's demonstrated capabilities, thereby supporting safe and effective clinical adoption.

## Clinical implications: trust, ethics, and impact

The cognitive alignment design implications extend well beyond usability. They resonate in ethics, safety, and fairness in healthcare. Trust follows once AI is aligned to human cognition. Trust is not blind faith in a machine; it's an interactive, reciprocal engagement (37). Trustworthy AI gains a place in the clinician's work flow not only because it's accurate, but because it's clear, explainable, and responsive to clinician input.

Interpretability at the cognitive level can enhance collective accountability. Clinicians who understand how and why a model makes a prediction will be more likely to use it responsibly. This is valuable in medico-legal contexts where accountability for decisions made in an AI system remains ambiguous (38, 39).

Additionally, cognitively compatible AI can be applied to eradicate health inequities (40). Vulnerable populations tend to be served poorly by opaque, one-size-fits-all models that disregard context. Where AI systems can explain to people in natural language and learn to respond to user input, they become fairer and more attentive to diverse populations.

Beyond trust and interpretability, AI in cardiovascular care must navigate complex ethical and legal obligations, particularly around patient confidentiality and data protection. Compliance with frameworks such as Health Insurance Portability and Accountability Act (HIPAA) in the United States or General Data Protection Regulation (GDPR) in Europe requires that AI tools not only safeguard identifiable health information but also ensure secure data transmission, storage, and processing (41). A key challenge lies in balancing the need for rich, multimodal datasets with the principle of data minimization (42). Potential avenues for overcoming these hurdles include the adoption of privacy-preserving techniques such as federated learning, homomorphic encryption, and differential privacy, which allow model training without exposing raw patient data (43). Embedding these safeguards into the design of AI tools can mitigate confidentiality risks while maintaining clinical utility, thereby fostering both ethical integrity and regulatory compliance.

## Discussion: reimagining cardiovascular AI as a cognitive partner

If AI is to earn its rightful place in cardiovascular medicine, it will have to shift from computational oracle to trusted cognitive counterpart—one that thinks alongside clinicians, not instead of them. That will require something other than additional data or deeper models; it will require a fundamental reimagining of AI's role in clinical reasoning (44).

Alignment of cognition is not an option—it's a medical necessity. Medicine, and especially where failure is calamitous, like in cardiology, is part interpretive art, part predictive science. Decisions aren't ever really made *in vacuo*; they're informed by accretive knowledge, real-time intuitions, and moral responsibility. Current systems, however accurate, will not succeed if they ignore this cognitive ecosystem (45).

This redescription upends several typical assumptions. As a first point, it challenges the typical assumption that accuracy is the be-all and end-all measure of AI quality. While accuracy may be a prerequisite condition, it's not at all sufficient (46). A model that performs well on retrospective data sets but is illegible or incompatible with day-to-day clinic practice is, in actuality, unusable. Practical utility depends on whether or not clinicians can interpret, question, and respond to the output of a model—rather than whether or not the model is predictive (47).

Second, it disrupts the automation narrative that still pervades much of AI discourse. Many clinical tasks should not—and cannot—be fully automated. Cardiovascular care involves empathy, dialogue, and deliberation—facets that no model can replicate. Instead of aspiring to replace human expertise, AI should aim to enrich it. It should enhance diagnostic confidence, reveal overlooked correlations, and support counterfactual reasoning (48). Recent evaluations of multimodal cardiovascular AI systems have shown that such capabilities are technically feasible, integrating imaging, physiological signals, and clinical data into unified, interpretable frameworks that improve both diagnostic accuracy and clinician trust (49). In short, it should amplify clinical cognition, not bypass it.

Third, it calls for an interdisciplinary design ethos. Cognitive alignment cannot be engineered in isolation by data scientists or AI specialists alone (36). Evidence from large-scale deployments in cardiovascular imaging supports this imperative, showing that the most successful implementations involve co-design between AI developers, cardiologists, and workflow specialists (50). It demands the collaborative insight of clinicians, behavioral scientists, cognitive psychologists, ethicists, and UX designers. The goal is not to make AI more “intelligent” in the abstract, but to make it more clinically intelligible in the real.

Cognitive alignment requires humility—recognizing that even advanced models are fallible and clinical expertise remains essential (51). This humility must be embedded into both design and deployment—through uncertainty quantification, contextual disclaimers, and mechanisms for clinician override.

Importantly, we must also recognize that cognitive alignment is not a static endpoint. It is an ongoing, iterative process—one that evolves with new data, new users, and new clinical realities (52). As AI systems are deployed across diverse settings—from tertiary hospitals to rural clinics—their ability to adapt to varying cognitive expectations will determine their impact and longevity. The development of structured evaluation frameworks for such adaptability has begun, with recent cardiology-focused AI research outlining domain-specific cognitive performance indicators and multi-level validation strategies (53).

To move forward, institutions and developers must embed alignment principles into every stage of the AI lifecycle. This means:

• Integrating cognitive walkthroughs into model validation;
• Designing user-centered interfaces that respond to differing cognitive loads and decision styles;
• Establishing feedback loops where clinicians can critique and correct model outputs;
• Creating metrics that measure usability, trust, and comprehension, not just performance curves.

**Figure 1:**
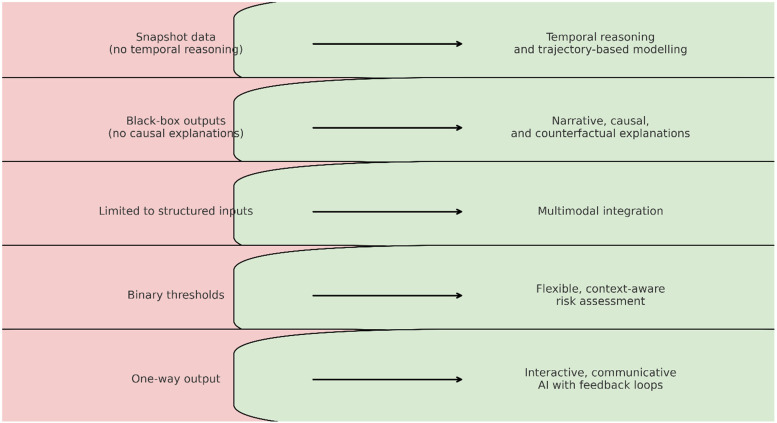
From current cardiovascular AI to cognitively aligned AI, illustrating the transformation from existing limitations to targeted solutions.

These shifts are not peripheral. They are central to the ethical deployment of AI in cardiovascular care. When clinicians and machines share a common cognitive ground, AI becomes more than a tool—it becomes a teammate. And in the complex, often ambiguous terrain of human health, that partnership may be the very thing that enables AI to finally deliver on its promise. Table 1 summarizes the main limitations identified in current cardiovascular AI, the proposed cognitive alignment approaches to address them, and the expected benefits of implementing these solutions. Figure 1 visually complements Table 1 by illustrating the transformation from current cardiovascular AI limitations to proposed cognitively aligned solutions, highlighting the direct mapping between gaps and targeted design improvements.

**Table 1:** Summary of current limitations in cardiovascular AI, proposed cognitive alignment strategies, and expected clinical benefits.

Theme	Current Limitation	Proposed Cognitive Alignment Solution	Model Scope	Concrete Method	Evaluation Metrics	Cardiology Use Case	Expected Benefit
Temporal Reasoning	Models trained on snapshot data ignore patient history and evolving trends	Integrate temporal reasoning and trajectory-based modeling	Longitudinal sequence models (RNN/LSTM/Transformer)	Rolling-origin evaluation, survival models (DeepSurv), temporal convolutional networks	Calibration/ECE, time-dependent AUC, missing-data robustness	HF progression monitoring	Aligns with clinicians’ longitudinal reasoning; improves relevance
Opaque Abstractions	Black-box outputs lack causal explanations	Provide narrative, causal, and counterfactual insights in natural language	Static tabular classifier + LLM	SHAP with summary plots + case-based exemplars, counterfactual generation	Clinician comprehension score, override appropriateness, decision-change rate	ACS triage	Enhances interpretability and trust
Disjointed Modalities	Limited ability to process multimodal, real-world clinical data	Support integration of structured, unstructured, and sensory inputs	Multimodal fusion models (tabular + imaging + text)	CLIP-style vision–language model, late-fusion neural architecture	Decision-curve net benefit, multimodal feature attribution	AF stroke risk prediction	Reflects the holistic data used in practice
Static Decision Boundaries	Fixed thresholds may ignore trade-offs, comorbidities, preferences, and changing context	Incorporate flexible, context-aware risk assessments	Probabilistic graphical models, calibrated classifiers	Conformal prediction intervals, Bayesian risk models	Calibration/ECE, net reclassification index	HF medication titration	Supports personalized and context-sensitive care
Lack of Interaction	AI tools operate as one-way output systems	Develop interactive, communicative AI that learns from user feedback	Interactive decision-support system	Active learning with clinician-in-the-loop feedback	Override appropriateness, learning curve slope, feedback incorporation rate	Post-PCI antiplatelet therapy planning	Promotes co-reasoning and shared decision-making
Gap in Deployment	Research advances not widely implemented in practice	Bridge research–practice gap through design focused on clinical workflow alignment	EHR-integrated CDS tool	SMART-on-FHIR integration, HL7-based audit logging	Time-to-decision, adoption rate, NASA-TLX workload score	AF anticoagulation initiation	Ensures real-world utility and adoption

## The reality gap: models in the lab, decisions in the clinic

Despite rapid advances in machine learning, the realities of clinical life are often absent from the AI development cycle. Most CVD models are trained in sanitized, structured environments—data is clean, outcomes are well-labeled, and populations are homogeneous (54). Yet hospitals are chaotic. Patients arrive late, symptoms evolve dynamically, data is often missing or conflicting, and clinicians must make fast, high-stakes decisions with imperfect knowledge.

This reality gap—between model assumptions and clinical conditions—is a key driver of cognitive misalignment (55). For example, a model may identify a patient as high-risk for heart failure based on elevated BNP and imaging data. However, in practice, the physician also notices signs of medication nonadherence, sleep apnea, or social factors like food insecurity—all of which are absent from the model's dataset, but critically affect outcomes and management (56).

A cognitively aligned model would flag its contextual limitations and allow space for human override, acknowledging that medicine is a narrative as much as it is a numeric function. Clinicians must not only predict events but also explain, persuade, and personalize care (57). AI models that ignore this context are not just incomplete—they're clinically unusable.

## Human factors and the design of cardiovascular AI

Borrowing from human factors engineering, AI systems should be evaluated not only for what they do, but for how humans interact with them under pressure, fatigue, and cognitive load. Studies in aviation and critical care show that even perfectly engineered tools fail when they misalign with human mental models.

In the context of CVD, this means designing interfaces and outputs that align with clinical thought processes. A risk prediction model embedded in an EHR should do more than just flag “elevated risk”—it should provide progressive disclosure, showing key contributing features, comparative examples (e.g., similar past patients), and options for simulation (e.g., “risk if blood pressure drops by 10 mmHg”).

Moreover, explanations must be tailored to user expertise. A cardiology fellow may want deep dive access to SHAP values and time-series feature trajectories, while a general practitioner may prefer high-level causal summaries. One-size-fits-all interpretability is neither effective nor scalable.

## Learning from failures: when alignment breaks down

There is growing evidence that even well-intentioned AI can mislead when cognitive alignment is lacking. In 2019, a large hospital system deployed a sepsis alert algorithm that was highly accurate in retrospective validation but triggered excessive false positives in real-time use. Clinicians quickly developed “alert fatigue,” leading to underuse—even in true positives. In other cases, such as cancer diagnostics or heart disease risk stratification, black-box tools have overemphasized non-clinical features (e.g., hospital location or scanner brand) because they correlate in training data, not because they matter pathophysiologically (58–60).

These failures are not purely technical—they reflect a cognitive design flaw**.** The models were not built to communicate uncertainty, adapt to clinician feedback, or explain themselves in actionable terms. When misalignment accumulates, trust erodes, and clinicians opt out of AI altogether (61).

This is not a rejection of AI—it is a plea for better design. When AI augments intuition rather than replacing it, clinicians feel empowered. When AI contradicts judgment without justification, clinicians resist. The success of cardiovascular AI hinges on this distinction.

## Beyond explainability: toward communicative AI

Explainability has become a buzzword, but too often it's reduced to technical jargon—“this feature had X impact on Y output” (8). True cognitive alignment requires more: not just explanation, but communication.

Communicative AI models engage users in a dialogue. They allow questions: “Why did you predict this?” “What would change your output?” “How does this compare to a similar patient last week?” This interactive paradigm transforms the model from a monologue machine into a clinical collaborator.

Such systems must also learn from users. When a clinician overrides a model repeatedly in similar cases, the system should learn to recalibrate or seek clarification. This is the foundation of human-in-the-loop learning, where models improve through shared judgment, not isolated optimization.

Imagine a scenario in a rural clinic. A junior physician uses a CVD risk model that suggests urgent referral. But the doctor knows this patient can't afford the trip, and instead opts for medication and monitoring. A communicative AI would register this decision, allow annotation, and use it to inform future outputs in similar contexts. This is not just personalization—it's localization of intelligence.

## Training for alignment: education and mindset

Creating cognitively aligned AI is only half the challenge. Clinicians must also be trained to interact with AI critically and constructively. This means embedding AI literacy into medical curricula—not just the mechanics of models, but the psychology of machine decision-making.

Clinicians must learn to ask:

• What assumptions does this model make?
• Is the data used representative of my patient?
• What happens when I disagree with the prediction?
• How do I explain this output to the patient?

These questions are not tangential—they are core to ethical clinical practice in the AI era. In cardiovascular care, where decisions often involve weighing long-term risks against immediate discomforts, these discussions are vital.

Moreover, institutions must cultivate a culture of dialogic AI use. Peer discussions, feedback loops, and governance structures should normalize the critique of model outputs and create shared accountability across teams. AI is not infallible—but used wisely, it can extend human capabilities.

## Cognitive alignment as a metric of success

Currently, most models are judged by technical metrics: AUC, precision, recall. But what about comprehension, adaptability, and user confidence?

We propose that AI models—especially in cardiovascular care—should be evaluated on a Cognitive Alignment Index (CAI), incorporating:

• Comprehensibility: Do clinicians understand why the model predicted this?
• Actionability: Does the output translate to a real-world clinical choice?
• Feedback receptivity: Can the model incorporate user feedback?
• Context awareness: Does the model recognize its own limits?
• Trust shift: Does use of the model increase, decrease, or recalibrate clinical trust?

Such metrics complement statistical ones, creating a more human-centered evaluation framework. Regulators, journals, and funders must expand their criteria to reward alignment, not just abstraction.

## Operationalizing the cognitive alignment index (CAI)

To enable systematic measurement, we propose a five-component CAI scoring framework (0–5 points per dimension; total range: 0–25, with higher scores indicating stronger cognitive alignment):

• Comprehensibility—accuracy of clinician responses to structured “explanation quizzes” with an answer key, testing understanding of why the model predicted X.
• Actionability—percentage of AI-driven recommendations resulting in guideline-concordant actions, attributable to the tool.
• Feedback Receptivity—measurable degree of model adaptation or refinement following structured clinician feedback during simulation or pilot deployment.
• Context Awareness—accuracy and frequency of self-reported out-of-distribution or uncertainty flags in live use.
• Trust Shift—change in clinician trust scores, measured pre/post using a validated trust-in-automation scale.

CAI validation plan includes:

• Construct validity: Correlate CAI scores with independent expert panel ratings of human–AI reasoning alignment in standardized case reviews.
• Predictive validity: Test whether higher CAI scores are associated with improved clinical endpoints (e.g., diagnostic accuracy, treatment appropriateness) and enhanced human–AI team performance (e.g., reduced decision latency, error interception rate).
• Reliability: Assess test–retest stability over repeated evaluations and inter-rater agreement when multiple evaluators score the same AI system.

This operationalization ensures CAI is not just a conceptual measure, but a reproducible, psychometrically robust index that can guide both research evaluation and real-world cardiovascular AI deployment.

To transition from conceptual framing to an actionable study design, we propose an evaluation blueprint incorporating both cognitive alignment and traditional performance metrics. In this design, clinicians interact with either simulated patient vignettes representing diverse cardiovascular scenarios or retrospective chart reviews from longitudinal EHR datasets, making decisions with and without AI support. Primary outcomes will include net clinical benefit via decision-curve analysis, calibrated Brier score for probabilistic accuracy, time-to-decision as an efficiency measure, and override appropriateness (beneficial vs. harmful overrides). Secondary outcomes will assess cognitive load using NASA-TLX, usability through SUS or UMUX-Lite, and trust calibration using validated pre/post trust-in-automation scales. For temporal reasoning, we will adopt explicit benchmarks: rolling-origin evaluation on longitudinal EHR data, robustness testing with controlled missing-data omissions, and distribution shift analyses across sites and time periods. This approach provides a structured, reproducible framework for evaluating cardiovascular AI on both technical merit and cognitive alignment.

## Broader systems implications: from product to policy

The call for cognitively aligned AI is not just a technical challenge—it is a policy imperative. Hospitals investing in AI must audit not only performance but adoption and experience. Health systems must ask: Are clinicians using this tool as intended? Are outcomes improving not just quantitatively, but qualitatively?

Medical liability frameworks must evolve to acknowledge shared agency between humans and machines. Who is accountable when an aligned AI tool offers sound advice, but it's ignored—or blindly accepted? Legal and ethical frameworks must reflect the co-decision nature of AI-supported care.

Further, equitable AI development demands diversity not only in data, but in design teams. Models aligned to one cultural or epistemic framework may alienate others. Cognitive alignment must extend to linguistic, regional, and professional diversity to avoid digital colonialism in global health.

While cognitively aligned cardiovascular AI can help mitigate rather than eradicate health inequities, risks such as automation bias, clinician deskilling, fairness–performance trade-offs, and distribution shift must be addressed. Counterfactuals and causal simulations offer value but face identification and transportability limits. Practical uncertainty methods—distributional calibration, conformal prediction, ensembles—are useful only if clinicians are guided on acting upon them in real decisions. Balancing benefits with these trade-offs is essential for safe and equitable adoption.

Cognitive alignment should also be embedded within regulatory, privacy, and adoption frameworks. Alignment metrics can complement existing Software as a Medical Device (SaMD) regulatory pathways by meeting transparency expectations, supporting post-market surveillance, and enabling auditability through EHR logging of AI-influenced decisions. Privacy-preserving training methods such as federated learning, differential privacy, and homomorphic encryption can protect patient data, but carry utility–privacy trade-offs, including potential accuracy loss, increased latency, and additional governance requirements. Lessons from early deployments underscore the importance of clear data-use agreements, integration into existing clinical workflows, and governance structures that maintain both technical performance and clinician trust. Practical uncertainty handling—through calibrated probabilities, conformal prediction intervals, or Bayesian credible intervals—can further ensure that AI outputs are communicated with appropriate confidence to support nuanced clinical decisions.

## Conclusion

The future of cardiovascular AI lies not in accumulating more data or building deeper neural networks, but in fostering greater understanding—between models and users, engineers and clinicians, prediction and meaning. Cognitive alignment is not a limitation; it is a catalyst for creating AI that is safe, trusted, and ethically grounded. When AI systems reason with us—narratively, temporally, and causally—they transcend the role of mere tools and become genuine partners in care. Achieving this vision requires designing models that reflect human reasoning, developing interfaces that encourage interaction, training clinicians to question rather than blindly follow AI, and evaluating systems based on their capacity for comprehension, not just calibration. In doing so, we shift AI from a silent oracle to an engaged collaborator, from a black-box enigma to an intelligible co-pilot. In the nuanced and high-stakes landscape of cardiovascular care, this shift could mean the difference between rejection and adoption, prediction and prevention, or even between a risk score and a saved life. Rather than asking only how accurate our models are, we must ask how well they think with us—because in medicine, success depends not just on what we know, but on how we think.
